# Changes in the landscape patterns of Black‐necked Crane habitat and its correlation with their individual population numbers during the past 40 years in China

**DOI:** 10.1002/ece3.10125

**Published:** 2023-06-12

**Authors:** Ying Yang, Chou Xie, Chaoyong Shen, Bangsen Tian, Shudong Wang, Xiaolin Bian, Yihong Guo, Yu Zhu, Haoran Fang

**Affiliations:** ^1^ Aerospace Information Research Institute Chinese Academy of Sciences Beijing China; ^2^ University of Chinese Academy of Sciences Beijing China; ^3^ Laboratory of Target Microwave Properties Deqing Academy of Satellite Applications Zhejiang China; ^4^ The Third Surveying and Mapping Institute of Guizhou Province Guiyang China

**Keywords:** Black‐necked Crane habitat, correlation analysis, habitat fragmentation, landscape index, landscape pattern

## Abstract

The landscape pattern of the Black‐necked Crane (*Grus nigricollis*) habitat in China changed at different spatial scales and long‐term periods due to natural factors and human activities, and habitat reduction and fragmentation threatened the survival of Black‐necked Cranes. The factors driving the habitat landscape pattern and individual population changes of Black‐necked Cranes remain to be studied. In this paper, based on remote sensing data of land use from 1980 to 2020, the changes in landscape pattern and fragmentation of the Black‐necked Crane habitat in China over 40 years were analyzed from two different spatial scales using the land cover transfer matrix and landscape index. The correlation between landscape and Black‐necked Crane individual population was analyzed. The most obvious observations were as follows: (1) Although transformation between landscapes occurred to varying degrees, the area of wetlands and arable land in the breeding and the wintering areas (net) increased significantly from 1980 to 2020. (2) Habitat fragmentation existed in the breeding and the wintering area and was more obvious in the wintering area. (3) The number of individuals of Black‐necked Cranes increased period by period, and habitat fragmentation did not inhibit their population growth. (4) The number of individuals of Black‐necked Crane was closely related to the wetland and arable land. The increasing area of wetlands and arable and the increasing landscape shape complexity all contributed to the growth of the individual population. The results also suggested that the number of individuals of Black‐necked Crane was not threatened by the expanding arable land in China, and they might benefit from arable landscapes. The conservation of Black‐necked Cranes should focus on the relationship between individual Black‐necked Cranes and arable landscapes, and the conservation of other waterbirds should also focus on the relationship between individual waterbirds and other landscapes.

## Graphical abstract

(1) This article quantifies the changes in landscape patterns of Black‐necked Crane breeding and wintering areas in China at different spatial scales, between 1980 and 2020. (2) This paper investigates the effects of changes in landscape patterns on Black‐necked Crane populations. (3) This paper verifies that the expansion of arable land in China does not threaten the survival of Black‐necked Cranes.

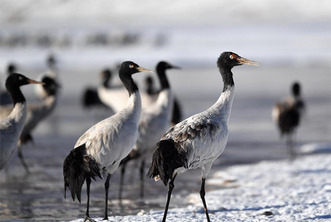

## 1 INTRODUCTION

Globally cranes generally have geographically separate breeding and wintering habitats, making it difficult to make protected areas that include both. Furthermore, many crane species also feed on anthropogenically modified land‐use types, mainly croplands after harvest (Liu et al., 2022; Mi et al., 2018). It is important to use remote sensing techniques to show how available habitat types have changed over time. In recent decades, the land‐use structure and landscape pattern of the plateau wetlands in China changed due to natural factors and human economic activities (Dong et al., 2020; Zhao et al., 2020), leading to the fragmentation of the habitats of the Black‐necked Crane (*Grus nigricollis*) and the reduction in suitable habitat areas (Li et al., 2022; Ru et al., 2019). In addition to limiting the movement, dispersal, and exchange of species, habitat fragmentation accelerates the extinction rate of endangered species and increases the loss of species diversity (Haddad et al., 2015; Newbold et al., 2015; Wilson et al., 2016).

Past literature has provided numerous assumptions regarding the relationships between crane habitat‐anthropogenic, and studies by several authors have demonstrated that human activities are detrimental to wild species (Bishop, 1996; Li & Li, 2005). However, numerous studies by scholars in Africa and South Asia have shown that agriculture is nevertheless of high value to cranes (Benn et al., 1995; Sundar, 2003). For example, most of the prey of the Black‐necked Stork in India is obtained from agricultural fields, and the Black‐headed Ibis also uses agricultural landscapes and densely populated urban landscapes, and Sarus Cranes in South Asia also live mainly in unprotected agricultural landscapes (Koli et al., 2019; Sundar, 2009, 2011a, 2011b). Within regions of high regional population density and cultivation, food diversity and density in arable land make foraging by cranes and storks closely associated with agriculture and can even affect their reproductive success. Moreover, it was also shown in China that Black‐necked Cranes use agricultural land and avoid forests in some areas (Han & Guo, 2018; Li, 2014; Tscharntke et al., 2005; Wang et al., 2019; Wu et al., 2020).

Many studies have been conducted on the link between habitat quality and landscape change for the Black‐necked Crane, and landscape pattern analysis methods were widely used because of their ability to reflect the habitat's environmental quality effectively (Liu, Ning, et al., 2018; Liu, Wilson, et al., 2018; Chu et al., 2018), the dynamics of habitat suitability (Abdolalizadeh et al., 2019), and the population response to habitat fragmentation (Steffens & Lehman, 2018). The choice of methods for habitat fragmentation analysis mostly favored quantitative analysis using multiple indices characterizing fragmentation and thus exploring the effects on birds (Guan et al., 2022; Steffens & Lehman, 2018), but the following aspects remain to be further investigated. First, analyses of long‐term habitat change processes are lacking. Second, there need to be more studies at large scales and research on the factors driving the Black‐necked Crane population at different scales. Third, there is a lack of validation of whether arable land expansion activities in China are detrimental to the survival of Black‐necked Cranes.

To address these issues, in this paper, we analyzed the long‐term habitat change and habitat fragmentation of the breeding and wintering areas of Black‐necked Cranes in China from 1980 to 2020 by using the land cover transfer matrix, landscape level, and class‐level landscape index. In addition, the number of individuals of Black‐necked Cranes was estimated, and the correlation between landscape pattern changes and the number of individuals of Black‐necked Cranes was analyzed. The analysis was conducted at the maximum spatial scale and with one of the typical wintering areas of Black‐necked Cranes (Caohai National Nature Reserve, Guizhou Province) for a more advanced study to show the factors driving changes in the number of individual Black‐necked Cranes at different study scales. We hypothesize and verify that agricultural landscapes in China are closely related to Black‐necked Cranes and that human activities are not always detrimental to the growth of Black‐necked Crane populations, and we will focus on changes in arable landscapes in addition to wetland landscapes.

## 2 STUDY AREA

**FIGURE 1:**
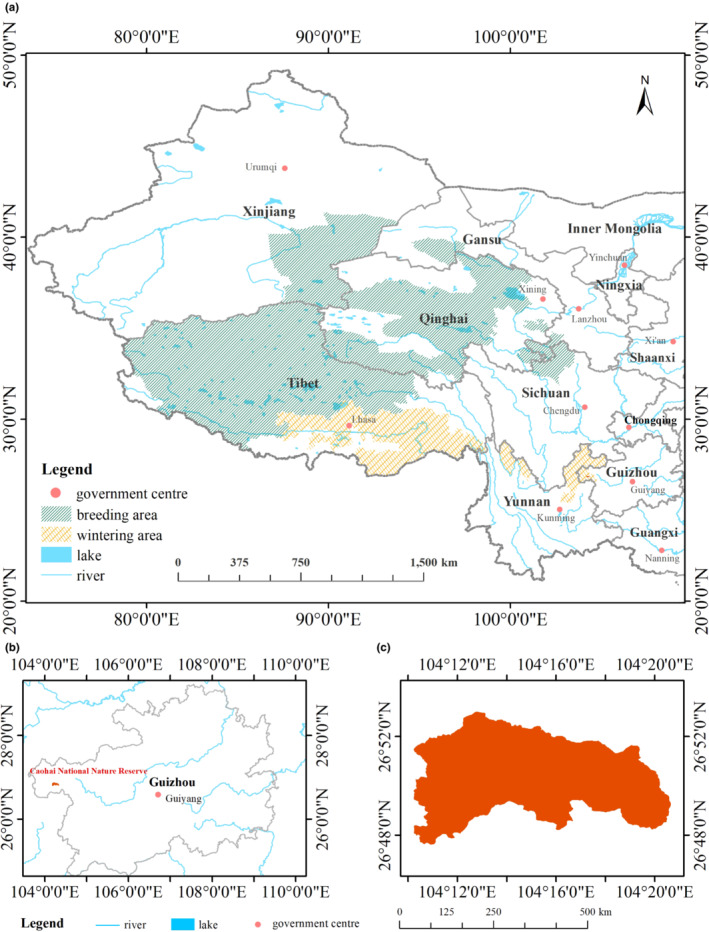
Distribution of Black‐necked Crane habitats in China. (a: The habitat range of Black‐necked Cranes in China, the green area represents their breeding area, and the yellow area represents their wintering area. b and c: Location of the Caohai National Nature Reserve).

The breeding areas of Black‐necked Cranes are located in Qinghai, Tibet, and the provinces of Sichuan that border Gansu and Qinghai (between 31° N to 39° N and 79° E to 104° E), and the wintering areas are located in the Yarlung Tsangpo River valley in southcentral Tibet, western and northeastern Yunnan, and western Guizhou (between 25° to 30° N and 87° to 105° E) in China (Hou et al., 2021; Li, 1999; Wang et al., 2020). The wintering sites and the breeding sites of the Black‐necked Cranes that were recorded during the study were included in the study area, which included 22 urban areas, as shown in Figure 1a (Kong et al., 2018; Li & Li, 2005; Li & Yang, 2003; Zheng & Wang, 1998).

In addition to the study of Black‐necked Crane habitats at large spatial scales, the Caohai National Nature Reserve (CNNR) in Weining County, Guizhou Province (between 104°10′ E to 104°20′ E and 26°47′N to 26°52′N) was selected as a typical study area since it has been a classic Black‐necked Crane wintering site (Figure 1b, c). The Caohai wetlands are one of the few natural reserves of subtropical highland wetland ecosystems in China. The natural landscape of the Caohai is severely damaged by intense human activities, resulting in a significant impact on the quality of Black‐necked Crane habitats, which caused fluctuations in their wintering population (Li, 2014; Ran et al., 2017; Tao et al., 1997). Therefore, understanding the changes in the landscape pattern of the Caohai can provide more targeted analyses of the effects of changes in the landscape pattern of the wintering habitat of the Black‐necked Crane on their population size.

## 3 METHODS AND MATERIALS

### 3.1 Data source and pre‐processing

The remote sensing data on land use in this paper were obtained from the Resource and Environmental Science Data Centre of the Chinese Academy (http://www.resdc.cn) (Gao et al., 2019; Liu et al., 2014, 2020; Liu, Ning, et al., 2018; Liu, Wilson, et al., 2018) at two different resolutions (1 km, 30 m, respectively), with 6 years of data for each resolution (for the years 1980, 1990, 2000, 2010, 2013, and 2020). The interpreted images used for remote sensing data on land use were mainly Landsat TM, and the accuracy of the interpretation was at least 85%. More data information can be found in Table S1. Except for the seven‐year interval from 2013 to 2020, the landscape pattern changes were analyzed at 10‐year intervals. The 1 km land‐use data were used to analyze the landscape pattern changes between the breeding and the wintering area at the large spatial scale and to compare the degree of habitat fragmentation in breeding areas with that in wintering areas. The 30 m land‐use data were used in the Caohai National Nature Reserve to analyze the long‐term habitat change and its impact on the population of Black‐necked Cranes at a small landscape scale in a more detailed way. The results of the index analysis at different landscape scales were not compared.

Black‐necked Cranes select inaccessible areas for breeding and wintering, such as swamps, lakeside meadows, reed swamps, and river valley swamps, which are far from human disturbance (Kong et al., 2011; Qian et al., 2009; Song et al., 2014). To investigate the effects of changing habitat landscape patterns on the Black‐necked Crane, the large‐scale regional classification was divided into six basic landscape types based on the ecological habits of the Black‐necked Crane in previous studies, including (1) open water areas (water body), (2) swamps (a type of wetland), (3) beaches, (4) arable land (paddy field and dryland), (5) built up areas, and (6) other land types (non‐wetland areas) (Table S2). In addition to the reclassification, wetlands (including open water areas, paddy fields, swamps, and beaches) and non‐wetland areas were also reclassified according to the Chinese land‐use classification standards (Ministry of Land and Resources of the People's Republic of China, 2017). This study assumed that Black‐necked Cranes could use wetlands and arable lands within the study range.

### 3.2 Analyses

The transfer matrix reflects the transformation process of the actual state of land cover types from the beginning to the end in the study area. The transfer matrix can be used to analyze the transfer direction and the number of transfers of different land‐use types. The generalized form of the land using a transfer matrix is shown in Equation 1:

(1) $$S_{\mathrm{ij}}=\left[\begin{array}{ccc} \begin{array}{c} S_{11}\\ S_{21} \end{array}\;\begin{array}{c} S_{12}\\ S_{22} \end{array} & \cdots & \begin{array}{c} S_{1n}\\ S_{2n} \end{array}\\ \vdots & \ddots & \vdots \\ \begin{array}{cc} S_{n1} & S_{n2} \end{array} & \cdots & S_{\mathrm{nn}} \end{array}\right]$$

where *S* represents the area, *n* represents the number of land‐use types, and *i* and *j* (*i* = 1,2,3…*n*, *j* = 1,2,3…*n*) represent the land‐use types before and after the transfer, respectively. *S*_*ij*_ represents the area of land‐use type *i* before transfer into land‐use type *j*.

Landscape index analysis is the most commonly used static quantitative analytical method to study the composition and characteristics of landscape patterns by analyzing the landscape pattern index. The landscape index includes the patch, class, and landscape levels. Among the various landscape indices, AREA_MN (mean patch area), LSI (landscape shape index), AI (aggregation index), COHESION (patch cohesion index), and SHDI (Shannon diversity index) can describe the size, shape, aggregation, and diversity of the landscape. It has been illustrated that these indices can affect bird populations (Adler & Jedicke, 2022). These indices have also been successfully applied in studies to quantify habitat fragmentation. AREA_MN, LSI, AI, and COHESION can be applied at the landscape and class levels, while SHDI can only be applied at the landscape level (Leng et al., 2022; Wang et al., 2014). Although there is a positive correlation between AI and AREA_MN and a strong positive correlation between AI and COHESION among these five indices (Li et al., 2020), all three indices were reserved for use in order to quantify habitat fragmentation characteristics and trends from three perspectives: area, connectivity, and aggregation. The landscape analytical software Fragstats 4.2 (McGarigal & Marks, 1995) was used to calculate the indices. The meanings of the indices are shown in Table 1.

**TABLE 1:** Landscape indices and ecological implications.

Index	Index description	Unit
AREA_MN (Mean Patch Area)	The average area of all patches or a particular type of patch in the landscape (Value range: AREA_MN ≥ 0). The smaller the average patch area, the more fragmented the patch.	Square kilometers
LSI (Landscape Shape Index)	This index reflects the shape complexity of the landscape (Value range: LSI ≥ 1). The closer the LSI is to 1, the simpler the overall shape. The larger the LSI, the more complex the shape.	None
COHESION (Patch Cohesion Index)	This index describes the degree of connectivity between patches and reflects the aggregation and dispersion state of patches in the landscape (Value range: 0 ≤ COHESION ≤ 100). The larger the value, the more aggregated the patch class and the stronger the physical connectivity of the patch class.	%
AI (Aggregation Index)	This index characterizes the degree of aggregation of a landscape or a patch class. Patch classes or landscapes with regular shapes and continuous distribution have higher AI values, higher internal connectivity, and lower fragmentation (Value range: 0 ≤ AI ≤ 100).	%
SHDI (Simpson's Diversity Index)	SHDI = 0 indicates that the entire landscape consists of only one patch, and an increase in SHDI indicates an increase in patch classes or a balanced distribution of patch classes in the landscape (Value range: SHDI ≥ 1). The larger the SHDI value, the higher the degree of fragmentation.	None

**FIGURE 2:**
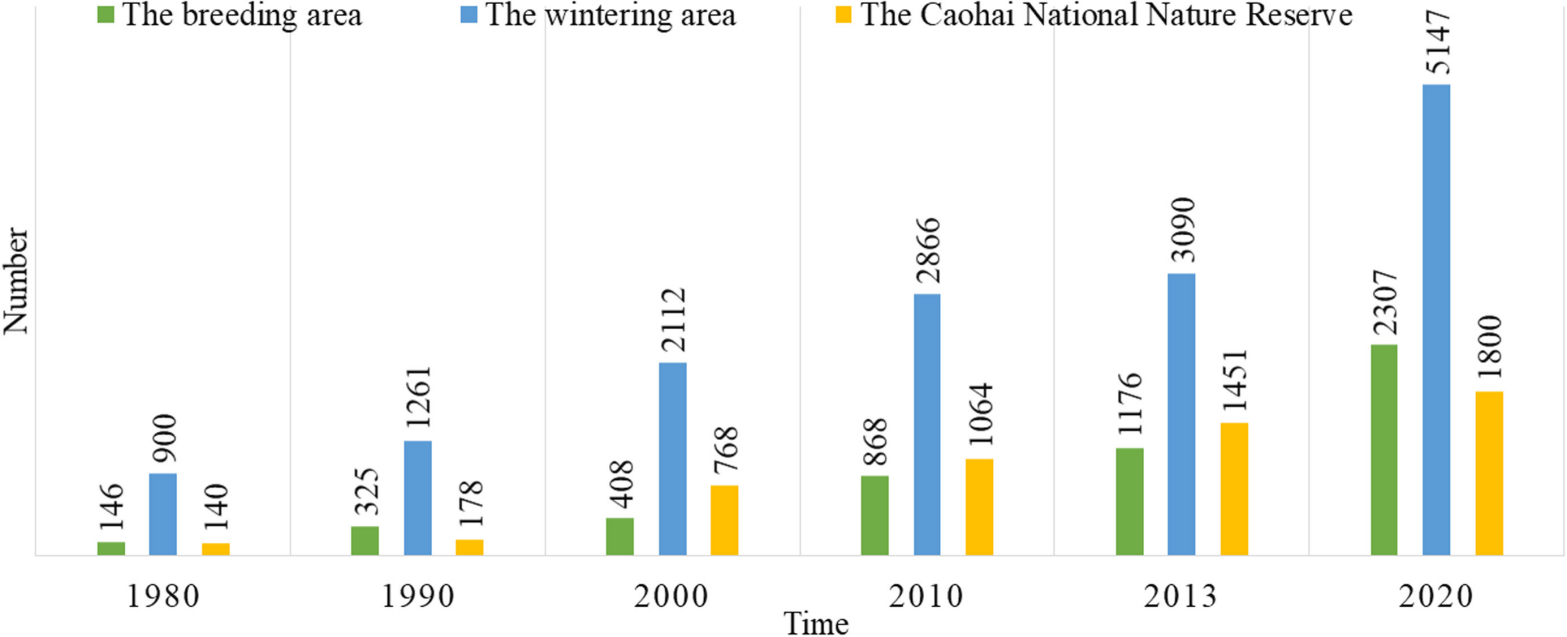
Summary of the annual number of individual Black‐necked Cranes from 1980 to 2020.

Although this study counted the number of individuals in each Black‐necked Crane distribution area in China since 1980, these records of Black‐necked Crane population distribution concentrated mainly in the Yunnan‐Guizhou plateau, the records of the Black‐necked Crane population in Tibet were relatively the rarest (Fang et al., 2020; Lu et al., 2017; Wu et al., 2020). Therefore, counting the number of individual Black‐necked Cranes in the wintering area was relatively easier. Due to the problem of missing and discontinuous data in some areas during the counting process, in this study, the total number of Black‐necked Cranes distributed in the breeding area of Yanchiwan National Nature Reserve of Gansu Province, Longbaotan Black‐necked Crane Nature Reserve of Qinghai Province, and Ruoerge Wetland Nature Reserve of Sichuan Province, which was within the breeding area, was used to represent the distribution of Black‐necked Cranes in the breeding area in China (minimum number), and the total number of Black‐necked Cranes distributed in the wintering area of Xundian Black‐necked Crane Reserve, Dashanbao Black‐necked Crane National Nature Reserve, Huize Black‐necked Crane National Nature Reserve and Caohai National Nature Reserve of Guizhou Province, which was included in the wintering area, was used to represent the distribution of Black‐necked Cranes in the wintering area in China (minimum number, Figure 2). In the statistical process, no consideration was given to whether individuals in the wintering and breeding areas were duplicated, and this paper focused only on the number of individual Black‐necked Cranes in different areas and the trend of population increase and decrease (Bishop & Li, 2002; Li, 1997; Li & Yang, 2003; Wei et al., 2021; Wu et al., 2019). The numbers of Black‐necked Cranes mentioned in the article are the total counted population.

IBM SPSS Statistics (26.0) software was utilized to calculate the Pearson correlation coefficient and its significance test between different landscape indices and individual numbers of Black‐necked Cranes at different scales. The results of the calculations were used to illustrate the habitat types used by the Black‐necked Cranes and the impact of the changing habitat landscape pattern on their population.

The results will be presented according to the study scale from large to small, in order of breeding areas, wintering areas, and Caohai National Nature Reserve. Moreover, analyses of land cover change, habitat fragmentation, and correlation between landscape and Black‐necked Crane population were analyzed for each area.

## 4 RESULTS

### 4.1 Landscape change analyses in the breeding and the wintering areas

Although the landscape domains of the breeding and wintering areas were woodlands, grasslands, and other landscapes (non‐wetland habitat types), the landscape details of the two areas differ significantly. The main features were that the breeding area accounted for a larger proportion of open water area, while the wintering area accounted for a larger proportion of arable land area (Table 2). The landscape patterns of both areas changed significantly after 2010, including a rapid increase in the open water area and the wetland (Table 2).

**TABLE 2:** Changes in the area of reclassified landscape types in the breeding area and the wintering area (unit: km^2^).

Region	Time	The open water area	Beach	Swamp	Arable land	Built up area	Wetland
The breeding area	1980	34,719	10,761	18,617	1907	331	64,099
	1990	33,977	10,476	18,741	1899	393	63,196
	2000	34,149	10,735	19,032	2040	451	63,918
	2010	34,718	10,786	18,805	2121	822	64,311
	2013	43,982	29,738	29,915	2442	905	103,638
	2020	47,448	29,441	29,435	2486	1287	106,327
The wintering area	1980	2619	906	69	14,147	163	5429
	1990	2642	894	72	14,008	177	5407
	2000	2665	872	71	13,985	208	5398
	2010	2659	874	71	13,940	253	5381
	2013	3966	1173	844	14,785	550	7728
	2020	4198	1169	824	14,671	828	9738

**FIGURE 3:**
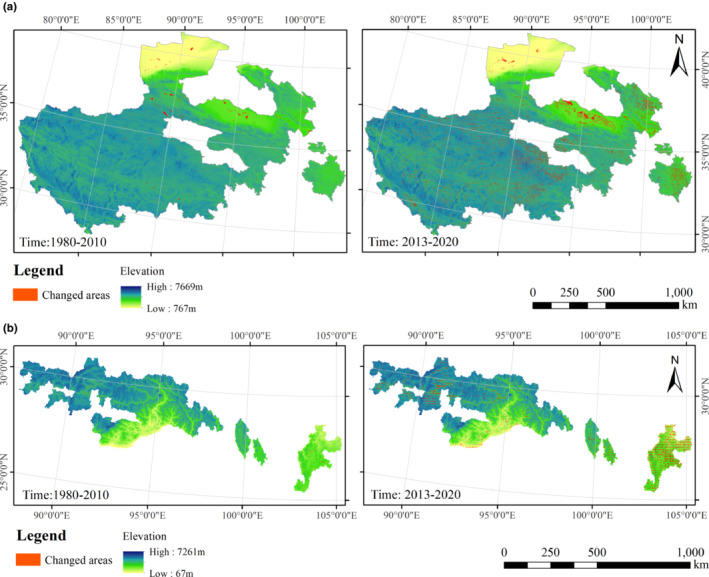
Distribution of areas with changing land cover in the breeding area and the wintering area (a: in the breeding area, b: in the wintering area).

The transfer matrix analysis results revealed that the primary sources of landscape area that changed in the breeding and the wintering area were other land types (non‐wetland habitat types). In general, the net wetland area increased, and there was a replacement of wetlands by other landscapes and transformation of other landscapes to wetlands in the process of land change in different periods, but the degree of transformation varied. More detailed matrix results can be found in the Appendix file (Table S3‐S6). Relative to the period from 1980 to 2010, the land‐use pattern within the two regions changed most significantly from 2013 to 2020. Land changes in breeding areas were concentrated in the east and north, and landscape changes in wintering areas were partially concentrated in the north (Figure 3).

The transitions of landscape area in the breeding and wintering areas were further summarized by the land cover transfer matrix (Table 3), and it can be seen that in the breeding area, other land types decreased the most. Built up area and arable land increased over time. Between 2013 and 2020, the area of open water area increased much more than other landscape types (by 3444 km^2^), and the source of the increase was mainly other land types (by 3090 km^2^), while the area of beaches and swamps also decreased extensively. In the wintering area, the significant reduction in arable land area in different periods was mainly transformed into other land types (non‐wetland habitat types), and the increase in open water area was mainly from the transformation of arable land and other land types (non‐wetland habitat types).

**TABLE 3:** Landscape transfer of the breeding area and the wintering area at different periods (unit: km^2^) (B: the breeding area, W: the wintering area).

Region	Time	Arable land	Built up area	Open water area	Beach	Swamp	Other land types
B	1980–2010	214	491	−1	25	188	−917
	2013–2020	37	384	3444	−297	−478	−3090
W	1980–2010	−207	90	40	−32	2	107
	2013–2020	−132	276	171	−4	−22	−289

### 4.2 Landscape index analyses for the breeding and the wintering areas

At the landscape level, the Black‐necked Crane habitat in the wintering area was more fragmented than in the breeding area, and the Black‐necked Crane habitat in the breeding area was better connected and more aggregated. However, after 2010, there was a trend of more obvious fragmentation of habitats in the breeding areas (Figure S1). At the class level, the shape of all patches in breeding and wintering areas tended to be more complex. However, the aggregation and connectivity of patches in breeding areas were generally better than those in wintering areas. Among all types of habitats, the connectivity and aggregation of open water areas in breeding and wintering areas were good, but there existed a trend of fragmentation, especially in the wintering areas. In addition, the connectivity and aggregation of arable land in the breeding area increased yearly. However, the connectivity and aggregation of arable land in the wintering area decreased (Figure S2). The index calculation results and more detailed analysis can be found in the Appendix S1.

### 4.3 Landscape change analyses in the Caohai National Nature Reserve

**FIGURE 4:**
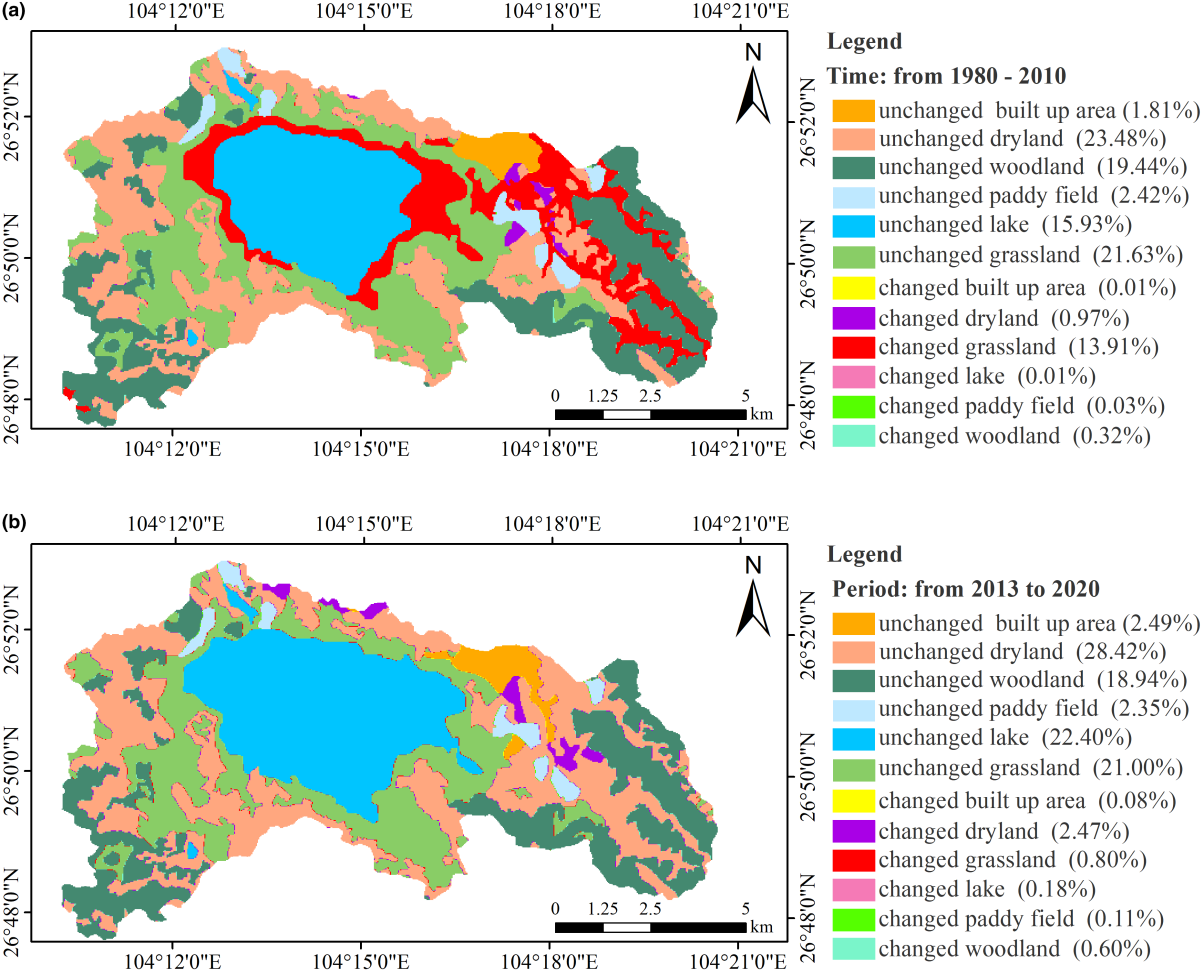
Changes of landscape pattern in CNNR from 1980 to 2020(The percentages in parentheses in the subplots indicate the proportion of the changed/unchanged area to the total area of the CNNR. For example, from 1980 to 2010, the unchanged grassland area accounted for 20% of the CNNR, and the changed grassland area accounted for 13.91%).

The landscape of the Caohai region includes the lake, paddy field, dryland, woodland, grassland, and built up area (Table 4). Before 2000, grassland was the dominant landscape category, followed by arable land. However, in 2000, the grassland decreased significantly, and the lake and wetland (net) area increased significantly. The built up area expanded rapidly in 2010 (Table 4), the landscape changes were mainly concentrated in the eastern and northern parts of the CNNR, and the map of changes in the landscape pattern of Caohai from 1980 to 2020 showed the expansion of the built up area to the southeast in the northeast (Figure 4).

**TABLE 4:** Changes in the area of landscape in the CNNR (unit: km^2^).

Time	Lake	Paddy field	Dryland	Woodland	Grassland	Built up area	Wetland
1980	16.10	2.48	24.72	19.86	36.02	1.84	18.58
1990	16.10	2.48	24.73	19.86	36.02	1.84	18.58
2000	22.79	2.48	24.72	19.86	29.34	1.84	25.27
2010	22.79	2.48	30.74	19.89	22.07	3.06	25.27
2013	22.80	2.49	31.30	19.82	22.04	2.60	25.29
2020	22.80	2.49	29.87	19.86	22.05	3.95	25.29

The land cover transfer matrix showed that the grassland and dryland landscapes changed the most in the CNNR (Table S7, Table S8). The landscape area transitions of the CNNR summarized by the land transfer matrix showed that the lake area increased the most from 1980 to 2010, followed by the dryland, while the grassland area decreased the most. The transformation between landscapes decreased from 2013 to 2020, the arable land area decreased the most, while the building area increased the most during this period (Table 5). More detailed results of the landscape transfer can be found in the appendix files (Table S7, Table S8).

**TABLE 5:** Landscape transfer area of the CNNR at different periods (unit: km^2^).

Time	Lake	Paddy field	Woodland	Grassland	Built up land	Dryland
1980–2010	6.69	0.00	0.03	−13.96	1.22	6.02
2013–2020	0.00	0.01	0.05	0.01	1.34	−1.41

### 4.4 Landscape index analyses for the Caohai National Nature Reserve

At the landscape level, the landscape connectivity and aggregation in the CNNR have been in good condition since 1980 with a balanced landscape distribution. However, the landscape shape was quite variable. From 1980 to 2000, the connectivity and aggregation of the Black‐necked Crane habitat in the Caohai area decreased, and there was a trend of habitat fragmentation. Although the landscape pattern improved after 2000, the landscape pattern was more fragmented in 2020 than in 2013 (Figure S3).

In the CNNR, the lake and arable land patches tended to be more complex in shape. The connectivity of all the landscapes in the CNNR was good, especially the lake, but the aggregation of the lake decreased slightly relative to the last century. After 2000, the connectivity and aggregation of drylands increased significantly, but in 2020 both connectivity and aggregation decreased than 2013 (Figure S4). The index calculation and more detailed analysis results can be found in the Appendix S1.

### 4.5 Correlation analyses between landscape pattern indices and the individual number of Black‐necked Crane

Habitat fragmentation existed in both breeding and wintering areas. However, the number of Black‐necked Cranes continued to increase, and habitat fragmentation did not significantly impact the number of individuals Black‐necked Cranes in the large study scale range. In the typical wintering area of Caohai National Nature Reserve, there was a weak positive correlation between the number of Black‐necked Cranes and landscape aggregation. The increased habitat aggregation may positively affect the number of individual Black‐necked Cranes at a small study scale range. Mainly from the landscape level perspective, the AREA_MN and COHESION index generally showed a negative correlation with the number of Black‐necked Cranes in the breeding and the wintering area. The gradually balanced landscape state (increased SHDI value) showed a correlation with the number of Black‐necked Cranes instead. The number of Black‐necked Cranes in CNNR increased with the degree of landscape aggregation and had a strong positive correlation with AREA_MN and SHDI (correlation coefficients were 0.755, 0.725) and a weaker positive correlation with AI index (correlation coefficient was 0.598) (Table S9).

In different study areas, different landscape indices were correlated with the number of individual Black‐necked Cranes to different degrees. However, whether in the breeding area, wintering area, or Caohai National Nature Reserve, the number of individual Black‐necked Cranes was positively correlated with the arable land and wetland. The number of individual Black‐necked Cranes increased with increasing areas of arable land and wetland, improving landscape connectivity and complexity of the shape (Tables S10, Table S11, Table S12). Wetland area had a vital role in influencing the habitat of Black‐necked Cranes (correlation coefficient ≥ 0.856), and the area of open water area correlated more closely with the number of Black‐necked Cranes than other patch classes (correlation coefficient ≥ 0.830) (Table S12).

At the large spatial scale, the arable patches in the breeding area were more closely correlated with the number of Black‐necked Cranes compared to the wintering area (correlation coefficient ≥ 0.835) (Table S10), which may be due to the foraging difficulty and foraging tendency of Black‐necked Cranes in the breeding and wintering areas. At small spatial scales, the increase in the number of Black‐necked Cranes was more obviously dependent on the lake (correlation coefficient was 0.868) and arable landscapes (correlation coefficients with paddy and dryland were 0.836, 0.841), and the increase in the connectivity of the lake and arable landscapes and the increase in the average patch size both contributed to the number of individual Black‐necked Cranes. In addition, the number of individual Black‐necked Cranes was not negatively affected by the increase of arable land in the Caohai area (Table S11, Table S12).

## 5 DISCUSSION

The habitat fragmentation was higher in the wintering area of the Black‐necked Crane. However, the habitat fragmentation trend existed in both breeding and wintering areas, especially in the open water area. During the process of land change in different periods, there existed the phenomenon that wetlands were replaced by other habitats and the transformation of other landscapes into wetlands. However, the net value of the total wetland area in breeding and wintering areas increased.

The connectivity and aggregation at the landscape level in the Caohai National Nature Reserve have been in good condition since 1980, especially for the lake. However, in 2000, the landscape pattern of the Caohai area changed, and the main body of the landscape was no longer grassland, and the substantial growth of the lake and arable land area reduced the grassland area.

In two research scales, the increase in area and complexity of the shape of the landscape of arable land and wetland were positively correlated with the number of individuals of Black‐necked Cranes. This study verified the hypothesis that Black‐necked Cranes are closely related to arable landscapes in China. The results of the study also suggested that the increase in agricultural expansion and human activities may not necessarily be detrimental to the survival of Black‐necked Cranes.

### 5.1 Analyses of the influencing factors of habitat landscape pattern change and landscape fragmentation at the large spatial scale

High landscape and habitat fragmentation in China are usually caused by anthropogenic disturbances, such as deforestation, agricultural reclamation, and urbanization (Dixo et al., 2009; Wu & Liu, 2014; Zhong et al., 2016). With the accelerated urbanization of breeding and wintering areas, the development of lakes for plateau development, the transformation of swamps, and the expansion of building sites and other human activities (Kong et al., 2018; Ruan et al., 2022) changed the landscape pattern of the Black‐necked Crane habitats, and impact of human activities on the landscape pattern was more evident in the wintering area (Kong et al., 2011; Li, 1996; Li et al., 2014). The degradation and disappearance of wetland landscapes exacerbate the fragmentation of suitable habitats for Black‐necked Cranes. However, policies (e.g., reforestation and wetland retreat) can cause landscape patterns to be affected by human activities to different degrees at different times (Kong et al., 2011), which also determines the degree of interconversion and transformation of wetland landscapes with other different landscapes.

The spatial heterogeneity of the landscape on a large scale is usually determined by climate and topography, with a general increase in breeding and wintering open water areas, which may be related to the warming and humidification in the northwest. In the context of global climate change, the Tibetan Plateau experienced extreme changes (Ma et al., 2017). Rising temperatures led to massive glaciers and permafrost melting, increased river runoff, and rising lake levels (Tang et al., 2019). Since the mid‐1980s, annual precipitation in the northwest has increased significantly, and temperature increases have accelerated (Gong et al., 2022; Shi et al., 2002; Zhang et al., 2019). The increased precipitation, higher temperatures, and the melting of glaciers and snowpack brought more runoff to rivers in the northwest, resulting in an increase in the areas of most lakes over the past 30 years (Fang et al., 2018). The results of the study also confirmed that the lake areas in both the breeding and wintering areas of Black‐necked Cranes tended to increase over the past 30 years.

### 5.2 Analyses of the influencing factors of habitat landscape pattern change and landscape fragmentation at the small spatial scale

Changes in the landscape pattern of the Caohai region have been strongly influenced by human activities. Especially before 1980, the natural landscape, such as wetlands in the Caohai area, was reduced due to over‐exploitation of agriculture and other human activities (Ran et al., 2017; Ru et al., 2019), which seriously affected the habitat of Black‐necked Cranes. The restoration and maintenance of the wetland landscape in the Caohai region benefited from the establishment of the Caohai National Nature Reserve after 1980, the implementation of the policy of returning farmland to wetlands and forests, and the increase in people's environmental awareness (Geng & Song, 1990; Wu et al., 2014), and the results of the study also indicated that the landscape connectivity and aggregation in the Caohai region have been in a good and relatively stable state since 1980. However, the expansion of the scale of building land in recent years reflects that the impact of human activities in the Caohai area is gradually deepening (Zhao & Yang, 2021). By keeping human activities within the carrying capacity of the reserve, the landscape pattern and ecology of Caohai can be continuously protected, thus ensuring the survival space of wintering Black‐necked Cranes.

### 5.3 Analyses of influencing factors for the correlation between landscape and the individual number of black‐necked cranes

The conversion of natural habitats to agricultural land was a fundamental cause of biodiversity loss (Tscharntke et al., 2005). However, a growing number of studies indicated that waterbirds such as Black‐necked Cranes might benefit from cultivated landscapes (Sundar & Subramanya, 2010). Black‐necked Crane subpopulations wintering in the Yarlung Zangbo River basin in Tibet, China, and the Dashanbao National Nature Reserve in Yunnan Province show a selection for farmland habitats (Tsamchu & Bishop, 2005). In the Caohai, Guizhou Province, arable land gradually becomes the main foraging site for wintering Black‐necked Cranes (Wu et al., 2020; Wu et al., 2021).

Studies by scholars in India and Africa have shown that Blue Cranes use agricultural fields and pastures (Allan, 1995), and Sarus Cranes and Brolgas also use treeless agricultural landscapes scattered in wetlands (Mukherjee et al., 2002; Sheldon, 2005; Sundar & Kittur, 2012). Breeding populations of woolly necked Storks are surprisingly high in the intensively cultivated and crowded areas of Jhajjar and Rohtak, India (Kittur & Sundar, 2021). The Black‐headed Ibis still maintain habitats in highly disturbed urban areas (Chaudhury & Koli, 2018; Koli et al., 2019; Sundar, 2006).

A possible reason for this phenomenon of closer spatial association of waterbird foraging sites with agricultural landscapes is that the shrinking and fragmentation of suitable habitat and foraging sites has led to the shrinking of food sources in natural landscapes, while food sources in agricultural landscapes support the survival of waterbirds such as the Black‐necked Crane (Han et al., 2018; Li, 1999; Yang et al., 2018), making the use of landscape patches by waterbirds such as the Black‐necked Crane has been altered.

Although the conservation of Black‐necked cranes should focus on cultivated landscapes, the wetland provides security and isolation from human disturbance (Li et al., 2022) and are an important basis for the survival of the Black‐necked cranes and other waterbirds. For example, in Indian mosaic landscapes with both agricultural components and natural wetlands, many waterbird species prefer natural wetlands (Sundar, 2004, 2006) and even avoid paddy fields (Maeda, 2001). Some waterfowl use natural wetlands to paddy fields in almost all seasons, and flooded agricultural lands do not adequately compensate for the lack of natural wetlands (Sundar, 2006).

Habitat fragmentation reduces waterbird habitat quality (Qi et al., 2014; Zou et al., 2014). However, in this paper, the results showed that the Black‐necked Crane population in China was not severely affected by habitat fragmentation, which may be related to the Black‐necked Cranes benefiting from increased arable landscapes, wetland landscapes, or perhaps the fragmentation level does not reach a threshold that poses a severe threat to the Black‐necked Crane population. The conservation of waterbirds should focus on landscape types closely related to individual waterbirds while ensuring essential wetland habitats.

## 6 CONCLUSIONS

Based on the land‐use data from 1980 to 2020, the spatial and temporal variation of the landscape pattern in the habitat of the Black‐necked Crane were revealed using the landscape index and the land cover transfer matrix. The correlation between the number of Black‐necked Cranes and the landscape pattern was investigated for different regions and scales in combination with the number of Black‐necked Cranes in different periods. It was verified that despite expanding arable land, the crane population increased in China. This study also filled a gap in recognition of the spatial and temporal change of the habitat of the Black‐necked Crane at different spatial scales and the correlation between the individual Black‐necked Crane populations and landscape patterns.

It was found that the increase in the wetland area, the complexity of landscape shape, and the reduction of human activities all contributed to the increase in the individual number of Black‐necked Crane, and there was a close relationship between arable land and Black‐necked Cranes. From the last century to the present, the landscape pattern of the Black‐necked Crane habitat has changed considerably, with shifts between different landscape types. Anthropogenic disturbances and climate change have differentially contributed to the changes in landscape patterns at different scales and regions. Policy, population, and economic development have accelerated changes in the landscape patterns, resulting in the fragmentation of the Black‐necked Crane habitat and reducing the range of suitable habitats. Although the number of Black‐necked Cranes has increased due to increased awareness and improved conservation measures, the number increase only partially represents the improvement in the quality of the Black‐necked Crane habitat probably means that Black‐necked Crane populations may only increase to a certain threshold. The fragmentation of habitat and the reduction of landscape types most relevant to the number of Black‐necked Cranes threaten the quality of Black‐necked Crane habitat. In addition, although the number of individual Black‐necked Cranes has increased, the melting of glaciers, increased rainfall, and changes in habitat environment caused by climate change may have an impact on Black‐necked Cranes, and the possible impact and the extent of the impact still need to be further investigated.

This study also shows that the individual number of Black‐necked Cranes is likely to continue to increase, and the downgrade of the conservation status of Black‐necked Cranes from “Vulnerable” to “Near Threatened” by the IUCN‐SSC is appropriate. For the conservation of waterbirds such as the Black‐necked Crane, while improving habitat fragmentation and strengthening policy guidance, we should also focus on the protection of the landscapes that are directly associated with the Black‐necked Crane. For example, protecting the arable landscapes in breeding areas within China and arable landscapes and lake landscapes within the Caohai National Nature Reserve. Conservationists should not assume that human activities are always detrimental to birds and should focus more on individual species and landscapes.

## AUTHOR CONTRIBUTIONS

**Ying Yang:** Conceptualization (lead); data curation (lead); formal analysis (lead); investigation (lead); methodology (lead); validation (lead); visualization (lead); writing – original draft (lead); writing – review and editing (lead). **Chou Xie:** Conceptualization (lead); data curation (lead); formal analysis (supporting); investigation (lead); methodology (supporting); validation (lead); writing – original draft (lead); writing – review and editing (lead). **Chaoyong Shen:** Conceptualization (lead); investigation (lead); supervision (supporting); validation (supporting); writing – review and editing (supporting). **Bangsen Tian:** Conceptualization (lead); supervision (supporting); validation (supporting); writing – review and editing (supporting). **Shudong Wang:** Conceptualization (lead); supervision (supporting); validation (supporting); writing – review and editing (supporting). **Xiaolin Bian:** Conceptualization (supporting); methodology (supporting); resources (supporting). **Yihong Guo:** Conceptualization (supporting); methodology (supporting); writing – review and editing (supporting). **Yu Zhu:** Conceptualization (supporting); methodology (supporting); writing – review and editing (supporting). **Haoran Fang:** Conceptualization (supporting); methodology (supporting); writing – review and editing (supporting).

## CONFLICT OF INTEREST STATEMENT

The authors declare that they have no known competing financial interests or personal relationships that could have appeared to influence the work reported in this paper.

## Supporting information


Appendix S1.

## Data Availability

The remote sensing data on land use in this study can be found in the figshare repository: https://doi.org/10.6084/m9.figshare.22214371.
