# CORRIGENDUM

**DOI:** 10.1002/ece3.10265

**Published:** 2023-07-12

In the recent article by Yang et al. (2023), the authors would like to add Chaoyong Shen as one of the corresponding author as shown below.

Correspondence

Chou Xie, Aerospace Information

Research Institute, Chinese Academy of

Sciences, Beijing, 100094, China.

Email: xiechou@radi.ac.cn

Chaoyong Shen, The Third Surveying and Mapping

Institute of Guizhou Province, Guiyang, China.

Email: whimfast@163.com

*Both corresponding authors have contributed greatly to the article.

The online version has been corrected online.
