# Supplementary material for: Changes in the landscape patterns of Black‐necked Crane habitat and its correlation with their individual population numbers during the past 40 years in China
Source: Ecol Evol. 2023 Jun 12;13(6):e10125. doi: 10.1002/ece3.10125 (PMC10261902; doi:10.1002/ece3.10125)
Supplement: Supplementary file 1 — Appendix S1. [file ECE3-13-e10125-s001.docx]

# Appendix

# S1. List of Supplemental Figures

## 1.1 Landscape level index analyses for the breeding area and the wintering area

At the landscape level, the wintering area showed a higher degree of landscape fragmentation, and the breeding area showed better connectivity, aggregation, more complex shapes, and higher diversity. The change in the value of the index can be divided into two periods, the period of relatively flat change (from 1980 to 2010) and the period of relatively significant change (2013 and 2020), with large differences in the values of the index in the two periods. Compared with the period from 1980 to 2010, in 2013 and 2020, the landscape connectivity has decreased, and the landscape aggregation in the breeding area has also decreased, but the aggregation of the landscape in the wintering area was slightly strengthened instead. In the breeding area, the trend of regional complexity of landscape shape is evident (Figure S1).

In recent years, a more obvious trend of habitat fragmentation existed in the breeding area. In the breeding area, the indices all showed a trend of increasing and then decreasing, indicating the process of increasing to decreasing landscape aggregation in the breeding area. In the wintering area, the landscape aggregation and connectivity were worse than those in the breeding area. The average area of patches was smaller, indicating that the habitat fragmentation in the wintering area was higher than that in the breeding area. Although the landscape pattern fragmentation in the wintering area decreased in 2020 relative to 1980 to 2010, the landscape pattern in 2020 remained fragmented relative to 2013 (Figure S1).

## 1.2 Class level index analyses for the breeding area and the wintering area

Patch aggregation and connectivity were generally better in the breeding areas than in the wintering areas. Patch aggregation was best in open water areas. The best connectivity was found for swamp patches, while the worst aggregation and connectivity were found for beaches. The aggregation of patches of arable land, beaches, and swamps was generally enhanced in 2020 than in 1980. Except for the fragmentation of the open water area, the aggregation and connectivity of the beach and swamp patches in 2020 decreased than that in 2013, showing fragmentation characteristics. The shape of all patches tended to be more complex, especially the beach and open water (Figure S2).

The results of the class index of the wintering area showed that the shapes of the various types of patches in the wintering area tended to be more complex, especially in the open water areas and swamps. Except for the arable patches, the shape of all types of patches was more regular than that of the breeding area. The patch connectivity of the wintering area was poor, and except for the swamp, the connectivity of all three types of patches tended to weaken, and there was obvious fragmentation. Compared with 1980, the patch aggregation of arable land, open water, the beach, and the swamp in 2020 showed a decreasing trend. The patch fragmentation was particularly evident in open water and beaches (Figure S2).


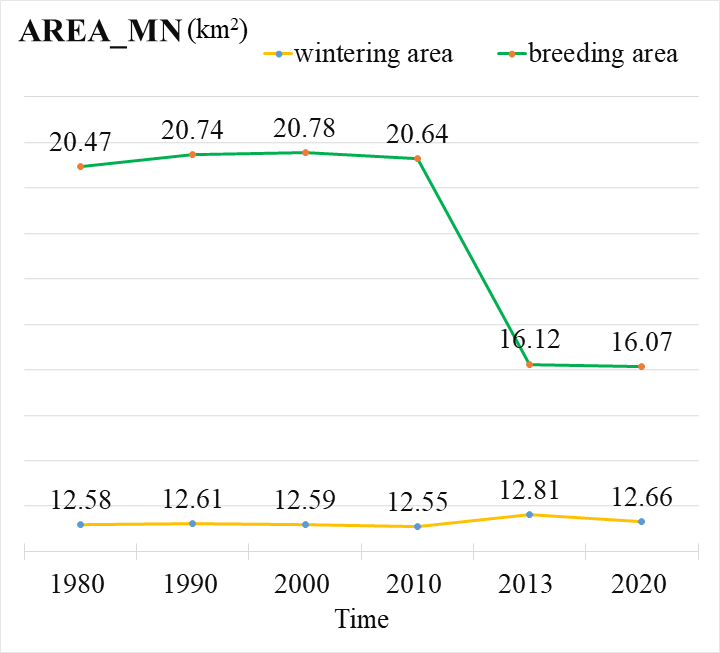

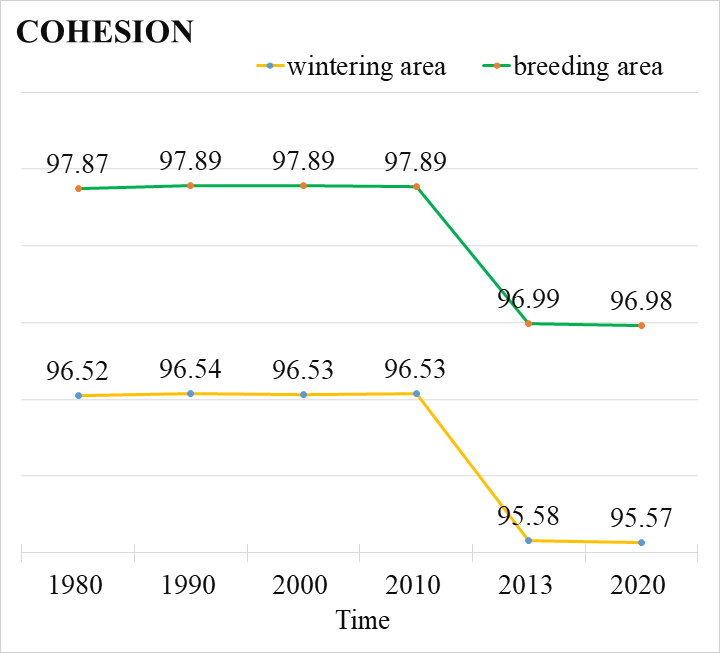


a b


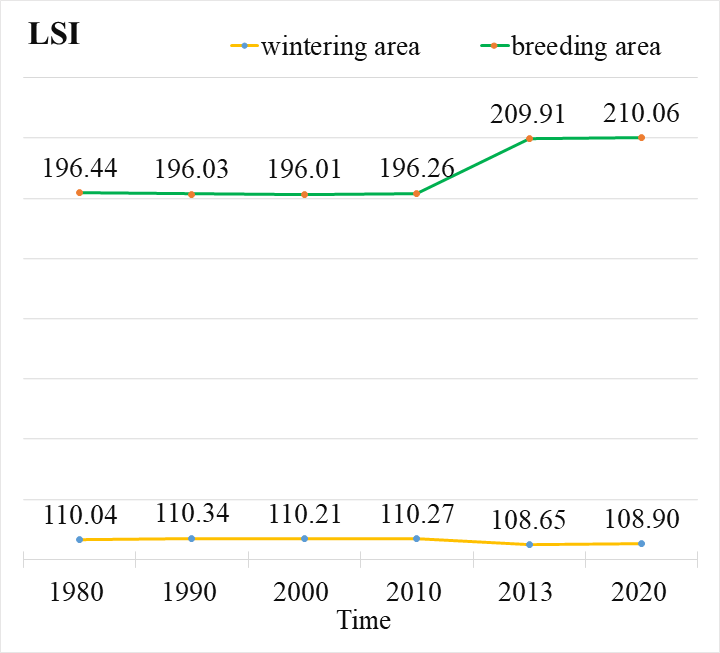

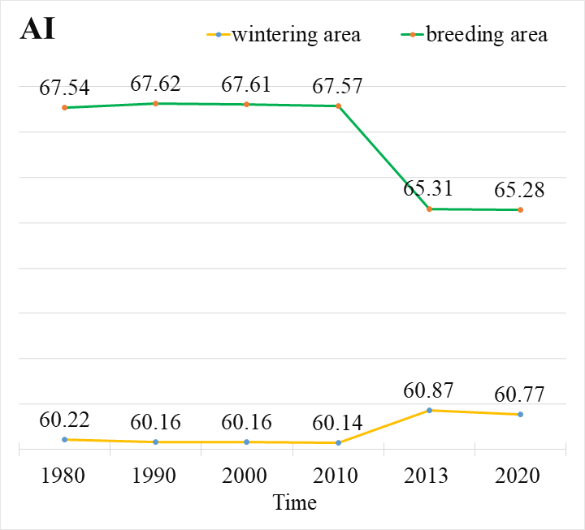


c d


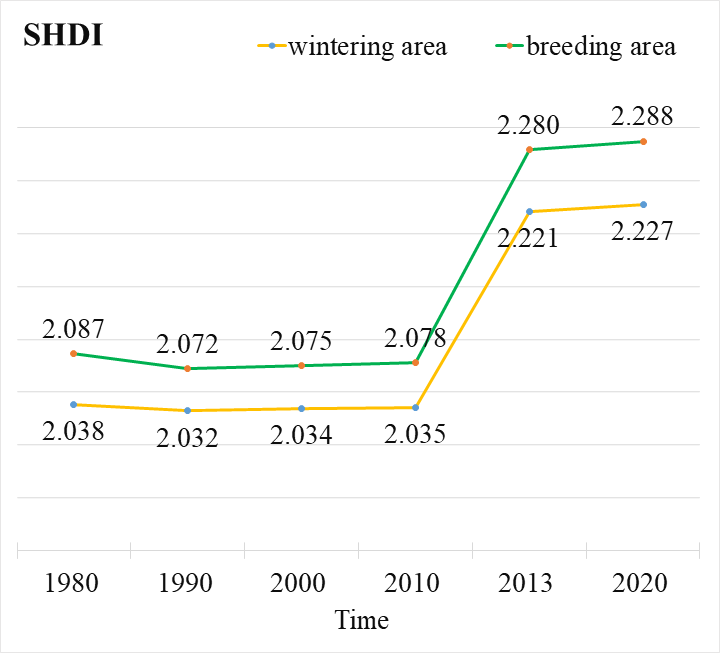


e

Figure S1 Calculated results of the landscape level index for the breeding and the wintering areas

(AREA_MN: The smaller the average patch area, the more fragmented the patch. LSI: The closer the LSI is to 1, the simpler the overall shape, and the larger the LSI, the more complex the shape. COHESION: The larger the value, the more aggregated the patch class and the stronger the physical connectivity of the patch class. AI: Patch classes or landscapes with regular shapes and continuous distribution have higher AI values, higher internal connectivity, and lower fragmentation. SHDI: The larger the SHDI value, the higher the degree of fragmentation.)


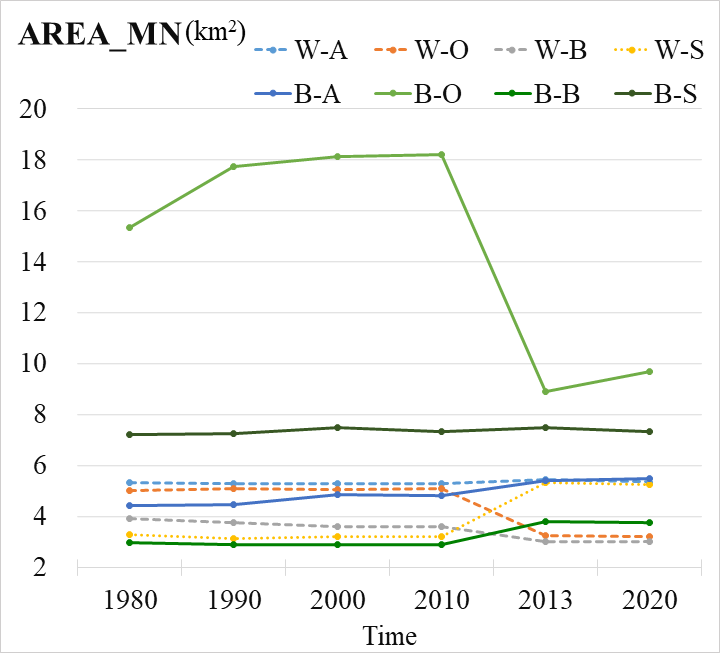

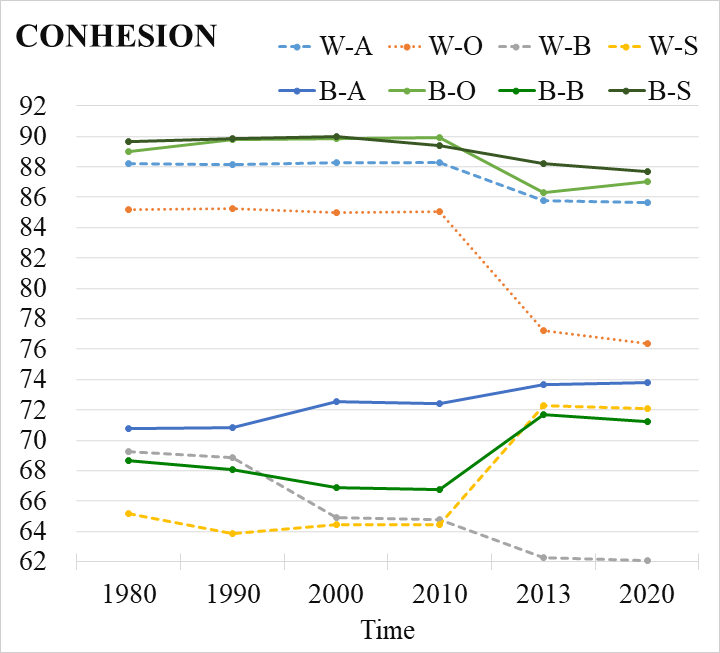


a b


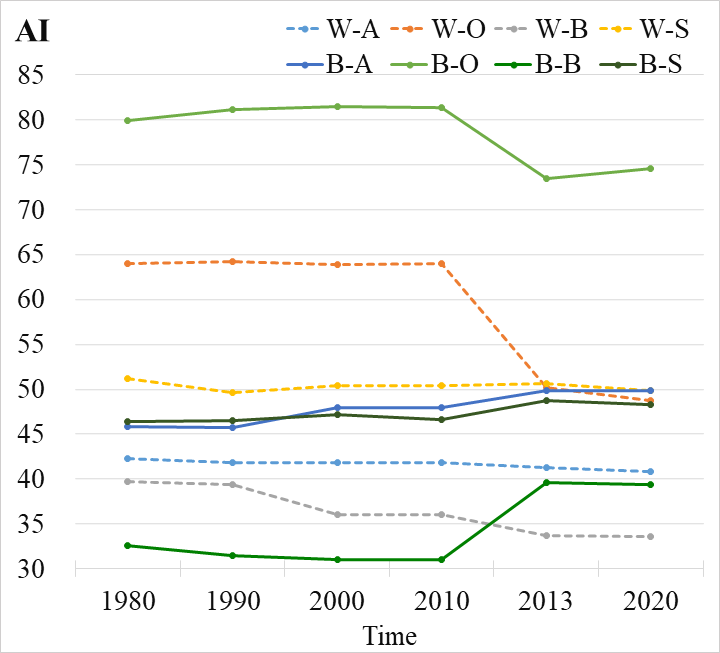

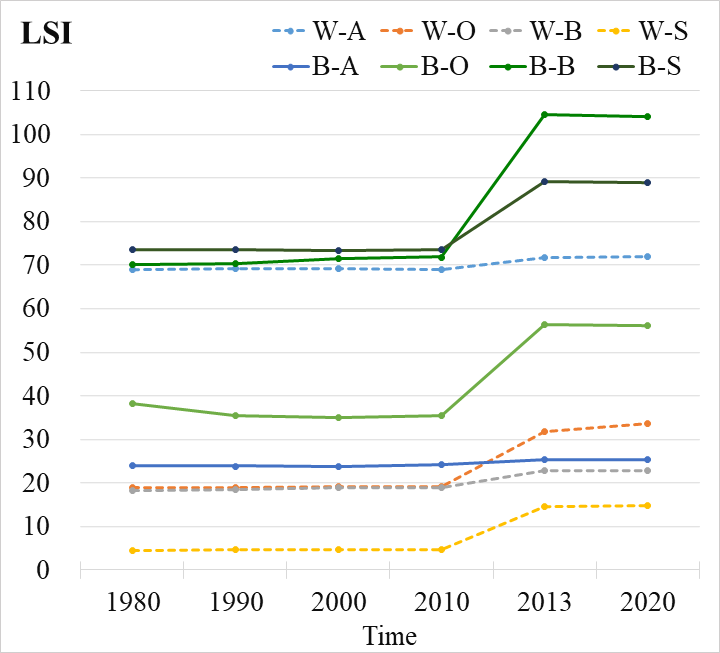


c d

Figure S2 Calculated results of the class level index for the breeding and the wintering areas

(The first capital letter represents the area (B, black-necked crane breeding area; W, the black-necked crane wintering area) and the second capital letter represents the class (A, the arable land, B, beach; O, open water area, S, swamp). AREA_MN: The smaller the average patch area, the more fragmented the patch. LSI: The closer the LSI is to 1, the simpler the overall shape, and the larger the LSI, the more complex the shape. COHESION: The larger the value, the more aggregated the patch class and the stronger the physical connectivity of the patch class. AI: Patch classes or landscapes with regular shapes and continuous distribution have higher AI values, higher internal connectivity, and lower fragmentation. SHDI: The larger the SHDI value, the higher the degree of fragmentation.)

## 1.3 Landscape level index analyses for Caohai National Nature Reserve

At the landscape level, the landscape connectivity and aggregation in the CNNR have been in good condition since 1980 with a balanced landscape distribution. However, the landscape shape was quite variable. Overall, the landscape pattern of the CNNR was the worst in 2000. From 1980 to 2000, the decrease in the mean patch area, connectivity, and aggregation, as well as the increase in SHDI values in the CNNR, indicated the existence of habitat fragmentation in the CNNR. Although the landscape pattern improved after 2000, the landscape pattern was more fragmented in 2020 than in 2013 (Figure S3).


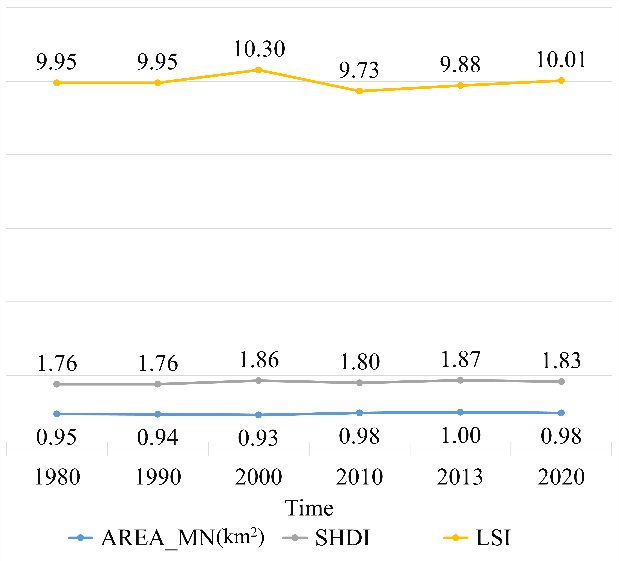

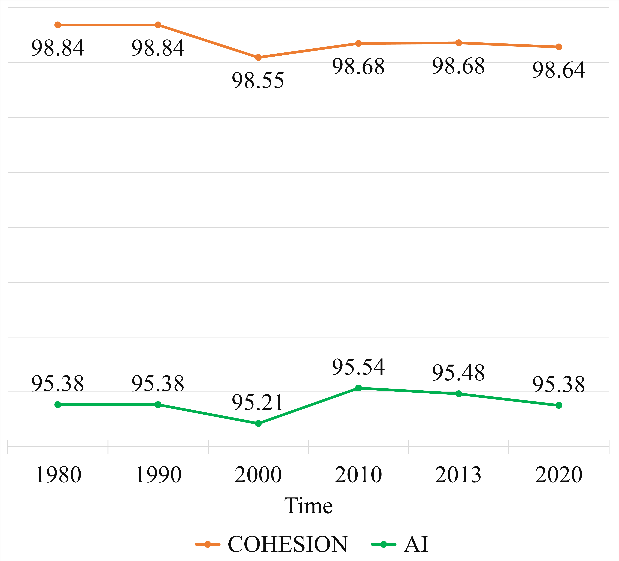


a. b.

Figure S3 Calculated results of the landscape level index for the CNNR

(AREA_MN: The smaller the average patch area, the more fragmented the patch. LSI: The closer the LSI is to 1, the simpler the overall shape, and the larger the LSI, the more complex the shape. COHESION: The larger the value, the more aggregated the patch class and the stronger the physical connectivity of the patch class. AI: Patch classes or landscapes with regular shapes and continuous distribution have higher AI values, higher internal connectivity, and lower fragmentation. SHDI: The larger the SHDI value, the higher the degree of fragmentation.)

## 1.4 Class level index analyses Caohai National Nature Reserve

In general, the shapes of the landscapes of all classes except grasslands tended toward complexity. The connectivity of all the landscapes in the CNNR was good, especially the lake (with AI and COHESION values > 99), which had the largest mean patch area and relatively most regular shape, but the aggregation of lakes decreased slightly relative to the last century. The mean patch area, connectivity, and aggregation of drylands increased after 2000. However, the mean patch area, landscape connectivity, and aggregation of the grassland, woodland, and built up area all decreased after 2000, showing a clear fragmentation (Figure S4).


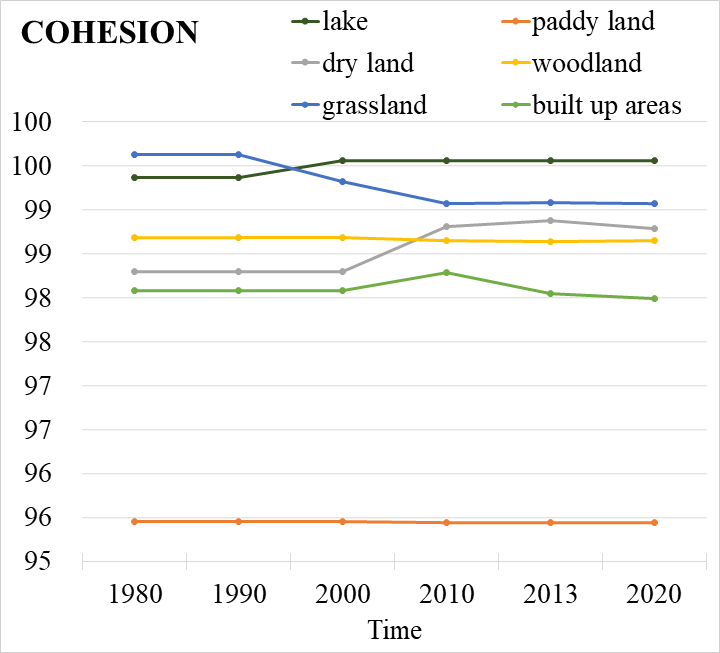

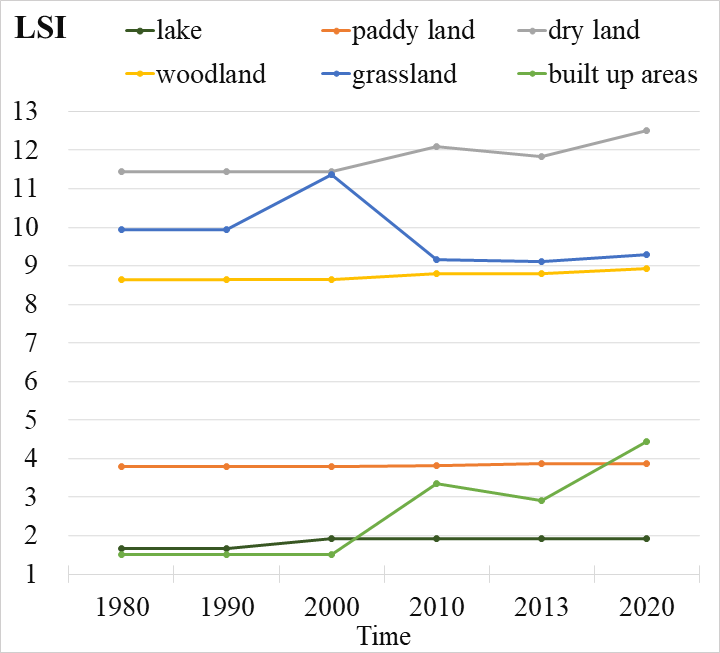


a b


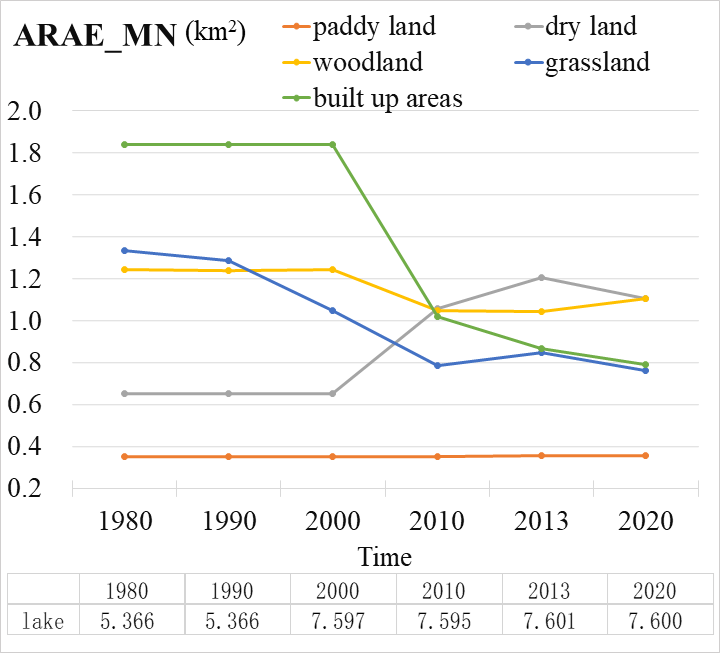

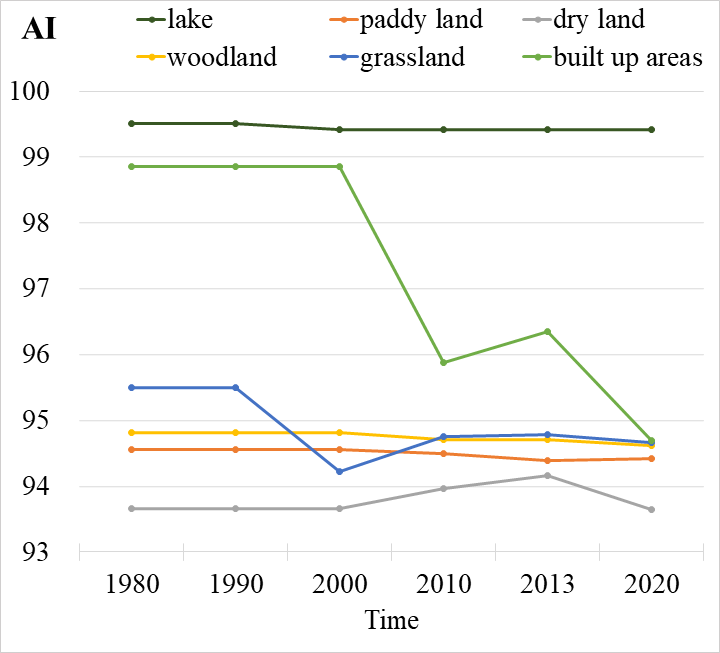


c d

Figure S4 Calculated results of the class level index for the CNNR

(AREA_MN: The smaller the average patch area, the more fragmented the patch. LSI: The closer the LSI is to 1, the simpler the overall shape, and the larger the LSI, the more complex the shape. COHESION: The larger the value, the more aggregated the patch class and the stronger the physical connectivity of the patch class. AI: Patch classes or landscapes with regular shapes and continuous distribution have higher AI values, higher internal connectivity, and lower fragmentation. SHDI: The larger the SHDI value, the higher the degree of fragmentation.)

# S2. List of Supplemental Tables

Table S1 Detailed parameters of the land use dataset

| **Region** | **Time** | **Resolution** | **Projection** | **Primary source images for interpretation** | **Classification accuracy** |
| --- | --- | --- | --- | --- | --- |
| **The breeding area and the wintering area** | 1980 | 1km | Albers normal-axis equal-area double standard latitudinal conic projection | Landsat-MSS，Landsat-TM | Data collection correct rate of arable land, urban and rural settlements is at least 95%, the correct rate of grassland, forest land, and water is not less than 90%, and the correct rate of unused land is not less than 85%. |
|  | 1990 | 1km |  | Landsat-TM |  |
|  | 2000 | 1km |  | Landsat-TM |  |
|  | 2010 | 1km |  | Landsat-TM |  |
|  | 2013 | 1km |  | Landsat-TM/ETM |  |
|  | 2020 | 1km |  | Landsat 8 |  |
| **The Caohai National Nature Reserve** | 1980 | 30m |  | Landsat-MSS，Landsat-TM |  |
|  | 1990 | 30m |  | Landsat-TM |  |
|  | 2000 | 30m |  | Landsat-TM |  |
|  | 2010 | 30m |  | Landsat-TM |  |
|  | 2013 | 30m |  | Landsat-TM/ETM |  |
|  | 2020 | 30m |  | Landsat 8 |  |

Table S2 Reclassification landscape types and the descriptions

|  | **Reclassified landscape types** | **Description** |
| --- | --- | --- |
| 1 | **Open Water Area**  (water body) | The new landscape type is derived from the merging of lake, reservoir pond, and river canal land cover types. |
| 2 | **Swamp** | The land with flat and low-lying terrain, poor drainage, long-term humidity, seasonal or perennial water accumulation, and the surface of which is covered with hygrophytes. |
| 3 | **Beach** | The tidal dip zone between the high and low tide levels of the coastal tide and the land between the high and low water level of the rivers and lakes during the flood and the normal periods. |
| 3 | **Arable land** | The new landscape type is derived from paddy field and dryland land cover types. |
| 4 | **Built up areas** | The new landscape type is derived from urban land, rural settlement, and other construction lands. |
| 5 | **Other land types**  (non-wetland habitat types) | Grassland, forest land, glacial tundra, and unused land are included, which do not directly affect the habitat selection of black-necked cranes. |

Table S3 Land transfer matrix in the breeding area of Black-necked Cranes from 1980 to 2010

(As the results of the matrix calculation only retained integer places, the matrix results had round-off errors, and the error area was less than 0.001% of the total area of the study area.)

| **1980**  **2010** | **Arable land** | **Built up area** | **Open water area** | **Beach** | **Swamp** | **Other land types** | **Total value (km^2^)** |
| --- | --- | --- | --- | --- | --- | --- | --- |
| **Arable land** | **1814** | 11 | 49 |  | 2 | 31 | 1907 |
| **Build up areas** |  | **330** |  |  |  | 1 | 331 |
| **Open water area** | 2 | 54 | **33219** | 150 | 138 | 1156 | 34719 |
| **Beach** | 4 |  | 221 | **10122** | 57 | 357 | 10761 |
| **Swamp** | 2 | 78 | 66 | 18 | **18208** | 245 | 18617 |
| **Other land types** | 299 | 349 | 1163 | 496 | 400 | **1347360** | 1350067 |
| **Total value (km^2^)** | 2121 | 822 | 34718 | 10786 | 18805 | 1349150 | 1416402 |

Table S4 Land transfer matrix in the breeding area of the Black-necked Cranes from 2013 to 2020

(As the results of the matrix calculation only retained integer places, the matrix results had round-off errors, and the error area was less than 0.001% of the total area of the study area.)

| **2013**  **2020** | **Arable land** | **Build up area** | **Open water area** | **Beach** | **Swamp** | **Other land types** | **Total value (km^2^)** |
| --- | --- | --- | --- | --- | --- | --- | --- |
| **Arable land** | **1620** | 48 | 18 | 18 | 2 | 726 | 2432 |
| **Build up areas** | 37 | **491** | 53 | 47 | 11 | 262 | 902 |
| **Open water area** | 12 | 6 | **37537** | 657 | 307 | 5414 | 43932 |
| **Beach** | 28 | 12 | 970 | **19158** | 318 | 9215 | 29700 |
| **Swamp** | 12 | 18 | 679 | 309 | **20055** | 8818 | 29890 |
| **Other land types** | 760 | 711 | 8120 | 9214 | 8720 | **1280040** | 1307565 |
| **Total value (km^2^)** | 2469 | 1285 | 47377 | 29404 | 29412 | 1304474 | 1414422 |

Table S5 Land transfer matrix in the wintering areas of the Black-necked Cranes from 1980 to 2010

(As the results of the matrix calculation only retained integer places, the matrix results had rounding errors, and the error area was less than 0.001% of the total area of the study area.)

| **1980**  **2010** | **Arable land** | **Build up area** | **Open water area** | **Beach** | **Swamp** | **Other land types** | **Total value (km^2^)** |
| --- | --- | --- | --- | --- | --- | --- | --- |
| **Arable land** | **13856** | 57 | 19 | 10 |  | 205 | 14147 |
| **Build up areas** |  | **163** |  |  |  |  | 163 |
| **Open water area** | 4 |  | **2604** | 2 | 1 | 8 | 2619 |
| **Beach** |  |  | 2 | **859** |  | 45 | 906 |
| **Swamp** |  |  |  |  | **69** |  | 69 |
| **Other land types** | 80 | 33 | 34 | 3 | 1 | **268803** | 268954 |
| **Total value (km^2^)** | 13940 | 253 | 2659 | 874 | 71 | 269061 | 286858 |

Table S6 Land transfer matrix in the wintering areas of the Black-necked Cranes from 2013 to 2020

(As the results of the matrix calculation only retained integer places, the matrix results had round-off errors, and the error area was less than 0.001% of the total area of the study area.)

| **2013**  **2020** | **Arable land** | **Build up area** | **Open water area** | **Beach** | **Swamp** | **Other land types** | **Total value (km^2^)** |
| --- | --- | --- | --- | --- | --- | --- | --- |
| **Arable land** | **10177** | 224 | 176 | 72 | 18 | 4014 | 14681 |
| **Build up areas** | 22 | **338** | 10 | 4 | 1 | 98 | 546 |
| **Open water area** | 117 | 13 | **2951** | 60 | 19 | 765 | 3925 |
| **Beach** | 65 | 13 | 72 | **750** | 12 | 244 | 1156 |
| **Swamp** | 14 | 7 | 26 | 10 | **604** | 180 | 841 |
| **Other land types** | 4081 | 227 | 860 | 255 | 166 | **259164** | 264753 |
| **Total value (km^2^)** | 14548 | 822 | 4096 | 1152 | 819 | 264464 | 285901 |

Table S7 Land transfer matrix of the CNNR from 1980 to 2010

(As the results of the matrix calculation only retained two decimal places, the matrix results had round-off errors, and the error area was less than 0.01% of the total area of the study area.)

| **1980  2010** | **Lake** | **Paddy land** | **Woodland** | **Grassland** | **Building land** | **Dry land** | **Total value(km^2^)** |
| --- | --- | --- | --- | --- | --- | --- | --- |
| **Lake** | **16.09** |  |  | 0.01 |  | 0.01 | 16.10 |
| **Paddy land** | 0.00 | **2.45** | 0.00 | 0.00 | 0.00 | 0.03 | 2.48 |
| **Woodland** |  | 0.00 | **19.63** | 0.04 |  | 0.16 | 19.83 |
| **Grassland** | 6.69 | 0.02 | 0.12 | **21.84** | 0.54 | 6.80 | 36.01 |
| **Building land** |  |  |  | 0.00 | **1.83** | 0.01 | 1.84 |
| **Dry land** | 0.01 | 0.01 | 0.11 | 0.16 | 0.69 | **23.71** | 24.69 |
| **Total value(km^2^)** | 22.79 | 2.48 | 19.86 | 22.06 | 3.06 | 30.71 | 100.96 |

Table S8 Land transfer matrix of the CNNR from 2013 to 2020

(As the results of the matrix calculation only retained two decimal places, the matrix results had round-off errors, and the error area was less than 0.01% of the total area of the study area.)

| **2013  2020** | **Lake** | **Paddy land** | **Woodland** | **Grassland** | **Building land** | **Dry land** | **Total value(km^2^)** |
| --- | --- | --- | --- | --- | --- | --- | --- |
| **Lake** | **22.62** | 0.00 | 0.00 | 0.15 |  | 0.03 | 22.80 |
| **Paddy land** | 0.00 | **2.37** | 0.01 | 0.02 | 0.01 | 0.08 | 2.48 |
| **Woodland** | 0.00 | 0.00 | **19.13** | 0.12 | 0.00 | 0.47 | 19.73 |
| **Grassland** | 0.16 | 0.03 | 0.14 | **21.21** | 0.02 | 0.47 | 22.01 |
| **Building land** |  | 0.00 |  | 0.04 | **2.52** | 0.04 | 2.60 |
| **Dry land** | 0.02 | 0.09 | 0.51 | 0.49 | 1.39 | **28.70** | 31.20 |
| **Total value(km^2^)** | 22.80 | 2.49 | 19.79 | 22.03 | 3.93 | 29.79 | 100.82 |

Table S9 The correlation between the landscape level indices and the individual number of Black-necked Cranes

（*P<0.05 level (two-tailed). **P<0.01 (two-tailed). B, the breeding area; W, the wintering area. C, the Caohai National Nature Reserve).

|  |  | **AREA_MN** | **LSI** | **COHESION** | **AI** | **SHDI** |
| --- | --- | --- | --- | --- | --- | --- |
| **B** | Pearson correlation | -.842^*^ | .845^*^ | -.846^*^ | -.846^*^ | .848^*^ |
|  | p-value (two-tailed test) | 0.036 | 0.034 | 0.034 | 0.034 | 0.033 |
| **W** | Pearson correlation | 0.385 | -0.701 | -0.784 | 0.707 | 0.791 |
|  | p-value (two-tailed test) | 0.451 | 0.121 | 0.065 | 0.116 | 0.061 |
| **C** | Pearson correlation | 0.755 | -0.106 | -0.664 | 0.275 | 0.725 |
|  | p-value (two-tailed test) | 0.083 | 0.842 | 0.151 | 0.598 | 0.103 |

Table S10 The correlation between the class level indices and the individual number of Black-necked Cranes in the breeding area and the wintering area

(*P<0.05 level (two-tailed). **P<0.01 (two-tailed). B, the breeding area; W, the wintering area.)

|  |  |  | **AREA_MN** | **LSI** | **COHESION** | **AI** |
| --- | --- | --- | --- | --- | --- | --- |
| **Arable land** | **B** | Pearson correlation | .882* | .904* | .835* | -.846* |
|  |  | P-value (two-tailed test) | 0.02 | 0.013 | 0.039 | 0.034 |
|  | **W** | Pearson correlation | 0.306 | 0.779 | -0.787 | -.926** |
|  |  | P-value (two-tailed test) | 0.555 | 0.068 | 0.063 | 0.008 |
| **Open water area** | **B** | Pearson correlation | -0.739 | .815* | -0.706 | -.846* |
|  |  | P-value (two-tailed test) | 0.094 | 0.048 | 0.117 | 0.034 |
|  | **W** | Pearson correlation | -0.79 | .818* | -.816* | -.812* |
|  |  | P-value (two-tailed test) | 0.061 | 0.047 | 0.048 | 0.05 |
| **Beach** | **B** | Pearson correlation | .817* | .850* | 0.681 | 0.797 |
|  |  | P-value (two-tailed test) | 0.047 | 0.032 | 0.136 | 0.057 |
|  | **W** | Pearson correlation | -.873* | .828* | -.890* | -.883* |
|  |  | P-value (two-tailed test) | 0.023 | 0.042 | 0.017 | 0.02 |
| **Swamp** | **B** | Pearson correlation | 0.164 | .840* | -.924** | 0.748 |
|  |  | P-value (two-tailed test) | 0.757 | 0.036 | 0.008 | 0.087 |
|  | **W** | Pearson correlation | 0.771 | 0.79 | 0.762 | -0.376 |
|  |  | P-value (two-tailed test) | 0.072 | 0.061 | 0.078 | 0.463 |

Table S11 The correlation between the class level indices and the individual numbers of Black-necked Cranes in the Caohai National Nature Reserve

(*P<0.05 level (two-tailed). **P<0.01 (two-tailed). ".^b^ " indicates that at least one variable is constant. In this study, the number of patches in lakes and paddy fields is constant. COHESION of the water field has remained almost unchanged since 1980.)

|  |  | **Lake** | **Paddy land** | **Dryland** |
| --- | --- | --- | --- | --- |
| **LSI** | Pearson correlation | .843* | .901* | .859* |
|  | P-value (two-tailed test) | 0.035 | 0.014 | 0.028 |
| **AREA_MN** | Pearson correlation | .855* | .836* | .887* |
|  | P-value (two-tailed test) | 0.030 | 0.038 | 0.018 |
| **COHESION** | Pearson correlation | .855* | .b | .862* |
|  | P-value (two-tailed test) | 0.030 |  | 0.027 |
| **AI** | Pearson correlation | -.821* | -.893* | 0.426 |
|  | P-value (two-tailed test) | 0.045 | 0.017 | 0.399 |

Table S12 The correlation between the wetland areas and the individual number of individual Black-necked Cranes

(*P<0.05 level (two-tailed). **P<0.01 (two-tailed). B, the breeding area; W, the wintering area, C, the Caohai National Nature Reserve.)

| **B** |  | **Wetland** | **Open water area** | **Beach** | **Swamp** | **Arable land** |
| --- | --- | --- | --- | --- | --- | --- |
|  | Pearson correlation | .863^*^ | .914^*^ | .840^*^ | .833^*^ | .907^*^ |
|  | P-value (two-tailed test) | 0.027 | 0.011 | 0.036 | 0.040 | 0.012 |
| **W** |  | **Wetland** | **Open water area** | **Beach** | **Swamp** | **Arable land** |
|  | Pearson correlation | .877^*^ | .830^*^ | 0.742 | 0.778 | 0.652 |
|  | P-value (two-tailed test) | 0.022 | 0.041 | 0.091 | 0.068 | 0.160 |
| **C** |  | **Wetland** | **Lake** | **Paddy land** | **Dry land** |  |
|  | Pearson correlation | .856^*^ | .868^*^ | .836^*^ | .841^*^ |  |
|  | P-value (two-tailed test) | 0.030 | 0.025 | 0.038 | 0.036 |  |
